# Inspection of wind turbine bolted connections using the ultrasonic phased array system

**DOI:** 10.1016/j.heliyon.2024.e34579

**Published:** 2024-07-14

**Authors:** Brandon Mills, Yashar Javadi, Farhad Abad, Saeid Lotfian, Charles MacLeod, Ali Mehmanparast, Gareth Pierce, Anthony Gachagan

**Affiliations:** a Centre for Ultrasonic Engineering, Department of Electronic and Electrical Engineering, University of Strathclyde, Glasgow, G1 1XQ, UK; b Naval Architecture, Ocean, and Marine Engineering, University of Strathclyde, Glasgow, G1 1XQ, UK; c Design, Manufacturing, and Engineering Management, University of Strathclyde, Glasgow, G1 1XQ, UK

**Keywords:** Non-destructive testing, Phased array ultrasonic transducers, Stress measurement, Bolt inspection, Ultrasonic testing

## Abstract

This study explores the inspection of bolted connections in wind turbines, specifically focusing on the application of Phased Array Ultrasonic Testing (PAUT). The research comprises four sections: Acoustoelastic Constant calibration, high tension investigation on bolts, blind tests on larger bolts, and Finite Element Analysis (FEA) verification. The methodology shows accurate results for stress while the bolt is under operative loads, and produces a clear indication of when it is above these loads and beginning to deform. PAUT emerges as a promising tool for bolt inspection, offering multiple imaging modes for simultaneous stress monitoring and defect detection. The study advocates for PAUT as a robust method to enhance wind turbine safety, longevity, and future in-situ testing.

## Highlights

• Industry practice for bolt load inspections utilises randomly placed strain gauges or single element ultrasonic transducers.
• This study proposes the utilisation of Phased Array Ultrasonic Transducers, which offer advantages over current methods.
• Phased array transducers can image around any defects and can provide a robust average from a single scan.
• Additionally, areas of plastic deformation can be identified by studying the Time of Flight distribution data.

## 1 Introduction

As a part of the movement towards renewable energy, wind turbines will become a vital part of our energy generation infrastructure [1]. An accurate and non-destructive inspection regimen is essential to optimise wind farms' lifecycle, especially in areas with high-stress concentrations to avoid early and unexpected failures [2,3]. One such location is in the flanged transition piece to the monopile foundation, where the stress concentration from the weight of the turbine above can cause bolts to loosen or even fail completely. These bolted connections all must adhere to specific standards, such as ASME PCC-1 and BS EN 14399, which provide safety and stability requirements [4]. Currently, inspection procedures require either randomly placed strain gauges for constant if incomplete monitoring [5,6] or manual inspection using Single Element transducers [7]. These methods each have drawbacks. In the case of strain gauges, placing them on each bolt is cost-prohibitive, hence the random placement [8,9]. Alas, this provides an incomplete picture of the overall stress of a given bolted connection point. Regarding the ultrasonic method, the deficiency is in the hardware. As Single Element transducers are used, only one A-scan is produced per scan, requiring multiple scans to build up a robust result. Ultrasonic stress measurement relies on the theory of Acoustoelasticity. First fully mathematically described by Murnaghan in 1937 [10], it states that the time of flight of an ultrasonic pulse through a material changes as a result of the material stress. In this context, the stress is caused by the axial tightening force originating from the nut [11]. Preload monitoring through the use of ultrasonic guided waves was first proposed by Yang et al. [12], and further, in depth studies have followed. Du et al. [13] utilised Finite Element Analysis (FEA) with experimental verification, a technique that will be used in this paper, to investigate torque loading on flange bolts. Xingliang et al. [14] proposed a bolt stress detection method utilising the energy ratio of multiple echoes, and by utilising this ratio instead of the attenuation coefficient, the interference of the guided waves on the detection signal was effectively addressed.

Javadi et al. [15] proposed a novel phased array methodology for inspecting bolts. This methodology has the advantage of scanning around any possible defects within the bolts through use of phased array probes, as well as being fully automated. This methodology has been tested manually also [16], and ties in well with other avenues of research such as the work of Dheeraj et al. [17] focusing on the remote monitoring of the scan results. In addition to the work of Javadi et al. which addresses imbedded defects within the bolt, Long et al. [18] have discussed dealing with impurities within the bolt affecting the ultrasonic scan. Smagulova et al. [19] discuss a methodology for safety assessment of dissimilar metal joins using phased arrays ultrasonic probes, and their layered inspection technique is effective at identifying layered interfaces. This is particularly useful within a wind turbine foundation, as the foundation, main body, and bolted connections can all be different materials, and any irregularities that this may cause can be addressed. Moles and Ginzel [20] demonstrated that PAUT probes can accurately detect and characterize internal defects, which are a major source of potential failure points for bolts. Finally, Yu et al. [21] proposed a methodology for high precision velocity measurement of reflected sound waves through analysis of the signal characteristics at layered interfaces. There are several new developments in the Phased Array system that make it a uniquely beneficial apparatus for bolt inspection. Though most Phased Array systems are unidirectional (the transmission and reception elements are in a straight line), two dimensional arrays, and more specifically two-dimensional circular arrays [22], are being investigated. These would allow for 3D volumetric stress analysis of bolted connections. Phased Array systems can also employ numerous unique algorithms [23] such as Full Matrix Capture (FMC) [24], which squares the number of acoustic paths available for analysis; Total Focusing Method (TFM) [25], which increases the focusing capability; and Phase Coherence Imaging (PCI) [26], which also reduces noise but utilizes the phase of the signals rather than the amplitude. These can all be combined to offer a robust, clear signal for analysis.

The Phased Array probe provides numerous advantages over the Single Element probe, which have been widely discussed. This research focuses on applying the principles of ultrasonic stress measurement to the Phased Array system, specifically to bolted wind turbine connections. It is shown in this paper that this methodology provides reliable measurements of the stress levels when a bolt is under operative loads, and a clear indication where a bolt has exceeded these loads. Application of this method will lead to a reduction in both premature bolt replacement and erroneous bolt acceptance, allowing wind turbines to extend their operative lifespan. Additionally, this methodology allows for simultaneous defect detection and stress measurement, demonstrated by Javadi et al. [15]. This greatly shortens the inspection process without sacrificing any rigor, reducing the amount of downtime a turbine will experience during the maintenance cycle.

## 2 Theory

**Fig. 1:**
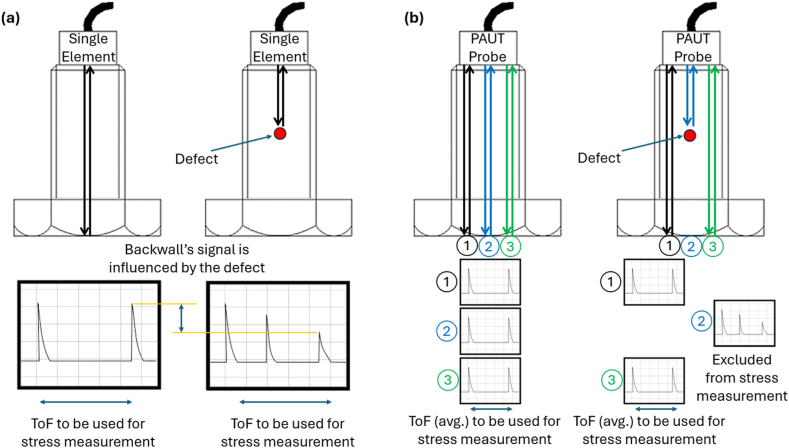
(a) A schematic single element inspection (b) A schematic phased array inspection.

This paper proposes a manual inspection procedure utilising PAUT probes that allows for simultaneous robust stress monitoring and defect detection in bolts. This procedure boasts several advantages over the current standard, the least of which being a minimal retraining requirement. A comparison is shown between a single element and a multiple element scan in Fig. 1 below.

In this figure, the red defect can be seen to block the user from obtaining a time-of-flight measurement when a Single Element probe, while the phased array probe can image around the defect, by considering only those elements where the defect is not encountered, saving time. It should be noted that only three acoustic paths are shown in the PAUT case, for simplicity. The PAUT imaging system has several benefits – the first clearly demonstrated in Fig. 1 above as simultaneous defect detection and stress measurement. Additionally, it offers versatility in its numerous imaging modes. One can perform a sector scan to “steer” the beam (allowing for inspection of the bolt threads), a linear scan to send the beam straight down, and in addition the Full Matrix Capture technique (FMC) can be utilised to increase the number of acoustic paths that can be analysed by considering every possible pairing of transmitter and receiver, instead of the standard pairings. This is particularly useful in this application, as a larger sample size will provide a more robust value for average stress and can also contribute to generation of further information – such as a stress or defect map.

The relationship between stress, material elasticity, the Acoustoelastic Constant, and time of flight is given by Equation (1):

(1) $$\Delta \sigma =\frac{E\cdot \Delta t}{A\cdot t_{0}}$$

where Δσ is the material stress, E is the Young's Modulus of the material, A is the linear Acoustoelastic Constant, $t_{0}$ is the time of flight in an unstressed material, and Δt is the difference between the time of flight in a stressed material and the same in an unstressed material. This is expanded in Equation (2) such that all *n* array elements are considered:

(2) $$\Delta \sigma =\left(\sum_{i=1}^{n}\frac{E\cdot \Delta t_{i}}{A\cdot t_{i0}}\right)/n$$

This equation provided a robust average stress value considering every available acoustic path. It is here where the Phased Array Full Matrix Capture technique provides a great advantage, as it allows for transmission and reception on every element, raising the number of available acoustic paths to the number of elements squared.

## 3 Methodology

### 3.1 Introduction

Four main experiments were performed as part of this study. The first was Finite Element Analysis, to aid in obtaining the expected average stress within the bolt under test. This was followed by a calibration to determine the Acoustoelastic Constant. This entailed a stress measurement performed on a bolt under standard hand tightening, using a strain gauge for verification. The third was a high preload inspection, performed on a clean bolt, to obtain a stress measurement under conditions that could be expected in an in-operation windfarm. Finally, a blind, in situ test was performed on an M42 bolt. These tests were performed using a Sonatest Detachable Active Array Head (DAAH) probe, with 20 elements, a pitch of 1.2 mm, and a centre frequency of 2.25 MHz. This setup was selected to maximise the penetration depth of the scan.

**Fig. 2:**
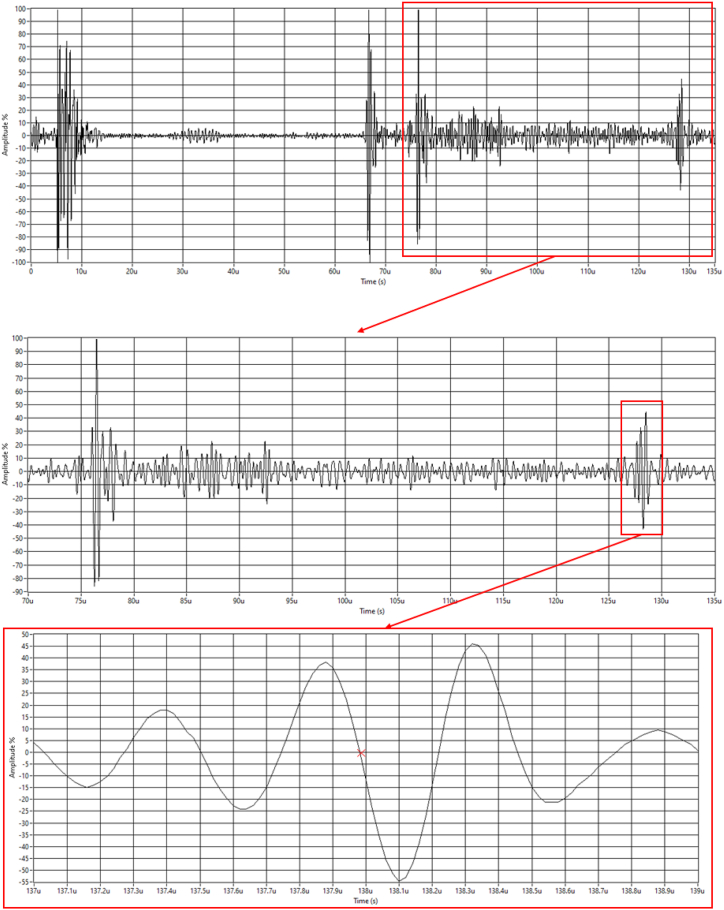
An example of A-scan data extraction. Top: The full A-scan. Middle: Zooming in on the second echo. Bottom: The backwall reflection, with the second zero crossing (the point where ToF data is extracted from) marked.

The inspection was performed according to the methodology of Mills et al. [16]: An initial sector scan is performed to identify defects both within the bolt volume and in the threads, and any that are found are compared against the acceptance criteria used. For the purposes of this paper, this was taken to be 10 % of the bolt diameter, or 3.6 mm. Any defects found above that size would allow the bolt to be classified as defective, and it would be marked for replacement. Following the initial scan, a second, more focused scan is performed to more accurately size any defects and gain an indication of their position within the bolt. This would be a linear B-scan inspection and would employ imaging techniques such as Full Matrix Capture (FMC) and the Total Focusing Method (TFM). The former is employed to increase the number of acoustic paths available for analysis, and the latter for noise reduction and to allow for more accurate defect sizing. At this point, the bolt can also be rejected if further defects are found that are either too large or too numerous to ignore, with the same criteria as above (10 % of the bolt diameter). If the bolt passes this stage, the probe is moved to a spot where the cleanest scan can be performed, a linear B-scan was taken, and the Time of Flight is measured on each acoustic path to be used for stress measurement using Equation 5. The backwall of the bolt was used as the reference point for time of flight extraction and, to account for the possible distortive effects of the coupling gel, the difference of the first and second echoes was used. This is demonstrated in Fig. 2. It is assumed that, as a matter of course, the ambient temperature whilst the inspection is being performed is known.

### 3.2 Finite element analysis verification

**Fig. 3:**
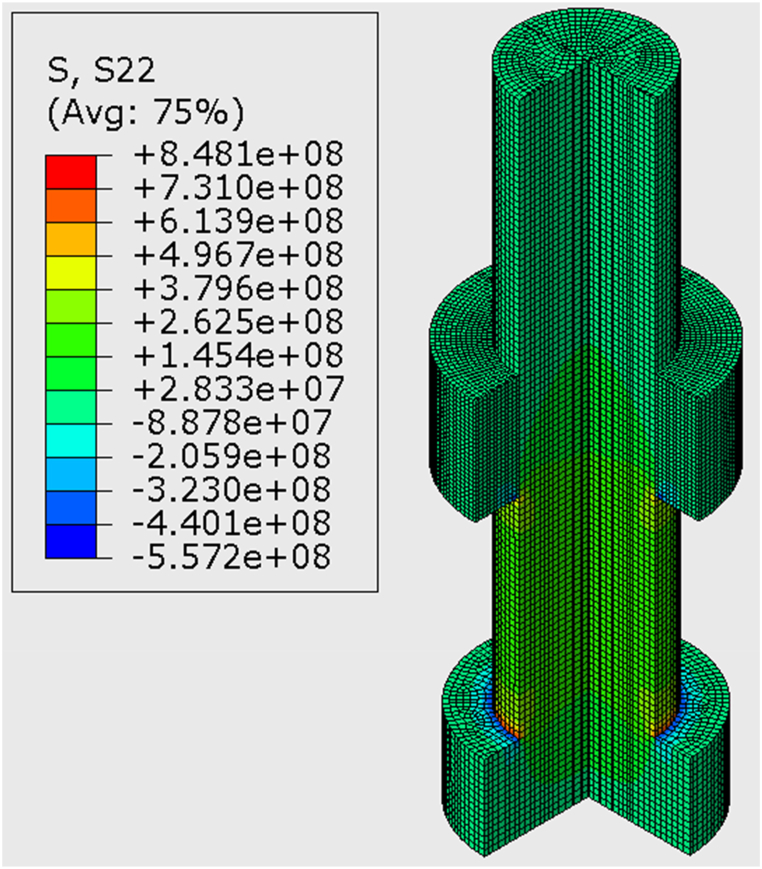
Finite Element Simulation for a bolt under 250 kN of force.

The finite element analysis (FEA) was conducted using the commercial software ABAQUS to enable a meaningful comparison between the stress measurements acquired through our ultrasonic methodology and those recorded by the washer-shaped load cell in the experimental setup. The modelling approach involved representing the bolt and nut as three-dimensional solid components, with the C3D8R element type (a 3D continuum stress/displacement element with reduced integration) employed for meshing. In the meshing process, we used sweep control to ensure better element distribution and improved mesh quality, which involved partitioning the bolt part to apply the technique. To simplify the model and focus on obtaining the average stress along the bolt's central path, the influence of the bolt threads was intentionally neglected. The position of the nut in Fig. 3 accurately reflects its initial position in the experimental setup.

The boundary conditions were carefully defined to replicate the experimental configuration accurately. Fixed constraints were applied to restrict all degrees of freedom at the contact surfaces between the bolt head, nut, plate, and washer. A tie contact constraint was also established between the bolt and nut contact surfaces, displaying their physical arrangement during the experiments. The bolt load applied in the FEA model was equivalent to the load cell data recorded during the experimental trials, ensuring a direct correspondence between the simulated and measured conditions. The average stress along the central path of the bolt was calculated for each level of preload, which in Fig. 3 was 250 kN. The FEA mesh and corresponding results, including stress distributions and average stress along the central path of the bolt, are presented in Fig. 3.

### 3.3 Acoustoelastic coefficient extraction

**Fig. 4:**
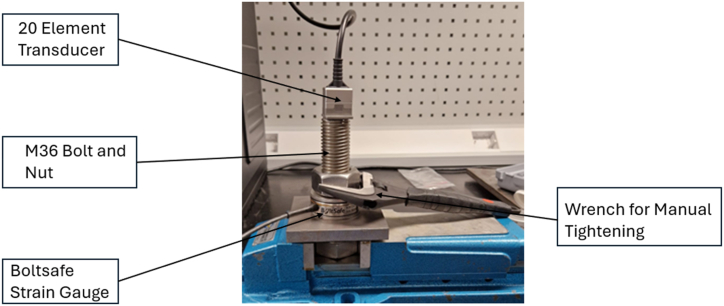
Experimental setup for Acoustoelastic calibration.

The bolt was set up as shown in Fig. 4. The preload was monitored using a Boltsafe strain gauge and the ultrasonic scanning by the before mentioned 20-element, 1.2 mm pitch PAUT probe with an elevation of 12 mm. The bolt was hand-tightened in a vice using an adjustable wrench. The experiment proceeded according to the description above, although no defects were expected, as a matter of good practice and in preparation for the high-tension experiment described in Section 3.4.

This experiment provided a solid grounding for the remaining ultrasonic experiments, as the Acoustoelastic Constant extracted was essential for analysis of the resulting data.

### 3.4 High tension investigation

**Fig. 5:**
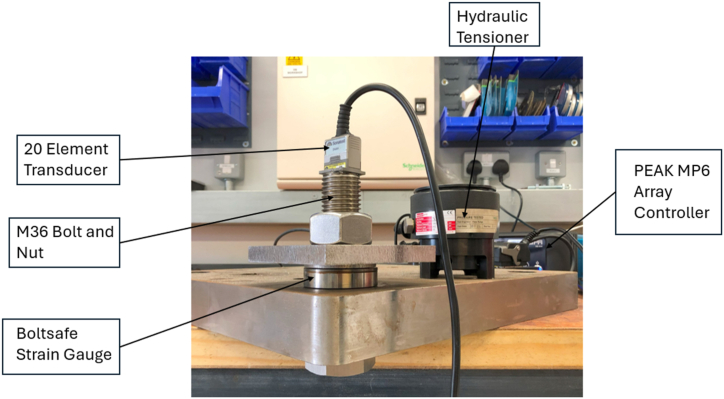
Bolt under inspection after hydraulic tensioning.

This experiment was performed using an M36 bolt. A tensioner was used to achieve appropriate levels of torque. Otherwise, the materials used were the same as the above experiment, excepting the lack of a vice (Fig. 5).

The PEAK Micropulse Controller system (MP6 HDR) was used to obtain the ultrasonic scans due to its versatility and High Dynamic Range (HDR), though for practical application of this methodology, a more compact system with a built-in screen would be beneficial for ease of transport. A clean bolt was specifically chosen for two main reasons: Firstly, safety – as it was to be tightened to 300 kN, any defects had the possibility of causing sudden failure. Secondly, as close to an operational standard, a bolt was sought for inspection, necessitating no defects and high tightening force.

### 3.5 Blind test on M42 bolt - towards the in-situ testing

**Fig. 6:**
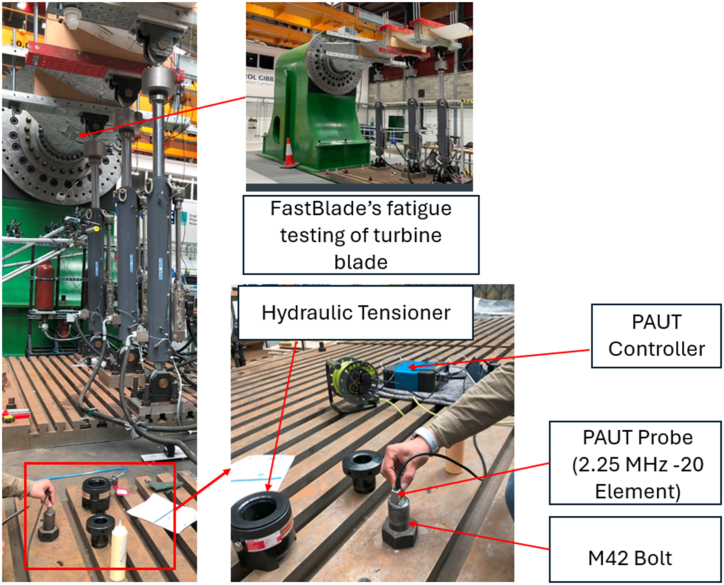
Finite Element Simulation for a bolt under 250 kN of force.

This section describes an experiment aimed at (i) evaluating the method's capability through a blind test (without the existence of calibration and laboratory preparations) and (ii) exploring the potential application of the approach for future in-situ testing. Consequently, a tidal turbine blade currently undergoing fatigue testing at FASTBLADE facilities is chosen to conduct the experiment (Fig. 6).

Within these facilities, there are large bolts (M42), the installation of which follows a similar procedure (using a hydraulic tensioner for tightening) to that of bolts utilised in the offshore wind turbine application—the primary focus of this paper. Considering the stringent requirements of the fatigue test for high accuracy and the involvement of dynamic loading, akin to the load cycles tolerated by offshore wind turbines, these bolts can effectively represent the comparable loading cycle and accuracy demands. It is noteworthy that a loose bolt can result in the failure of the fatigue test, mirroring the criticality observed in the connection of the monopile and transition part in offshore wind turbines, potentially leading to turbine failure if not properly secured. The tested M42 bolt was longer than any previously tested, and was inspected under an in situ load and zero load to ensure that this system could be adapted on the fly to detect any difference in time of flight in different bolt sizes.

## 4 Results

### 4.1 Finite element analysis

**Fig. 7:**
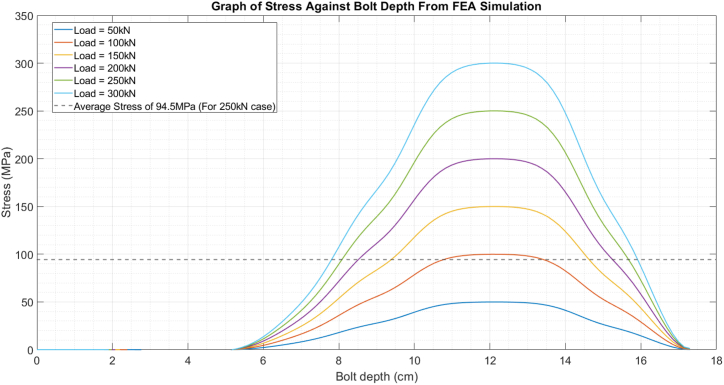
Load distribution graph from Finite Element Analysis, with the average stress when load is 250 kN indicated by the dashed line.

Finite Element analyses were used in these experiments to provide a baseline and expected average stress for comparison with our findings. This was achieved by performing the FEA at the various loads (50–300 kN) and then taking the average of the resultant stress across the central path of the bolt. For instance, when the load cell indicates 250 KN, the maximum stress is 250 MPa, while the average stress is only 94.53 MPa, as shown in Fig. 7. Therefore, it is crucial to simultaneously consider the average stress and verify the ultrasonic results through FEA and ultrasonic stress measurement.

### 4.2 Acoustoelasticity

**Fig. 8:**
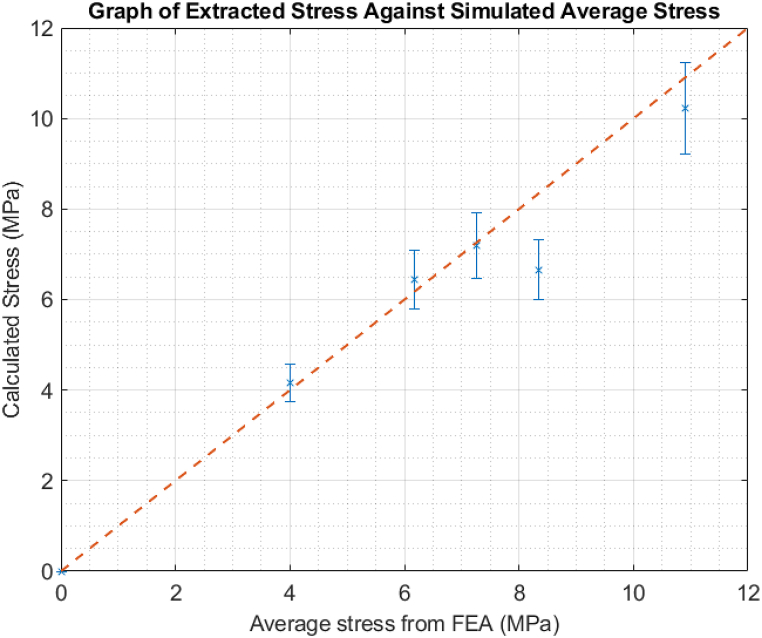
Graph of stress extracted from data against Finite Element simulated average stress. As expected, it makes a linear fit.

The Acoustoelastic Constant was determined by comparing the stress extracted from the measured time of flight values and the average stress from a finite element analysis. This is due to the washer-shaped load cell only measuring peak stress, and not the average stress of the bolt. Equation (1) was solved for A such that a line of best fit with a gradient of one passed through most of the points. This can be seen below in Fig. 8:

From this experiment, an Acoustoelastic Constant of $7.9\times 10^{6}$ was obtained for use in the following experiments. Due to the manual aspect of this experiment, only a few values could be obtained. It must be noted that this calibration is applicable only to this material, and would need to be repeated for any other alloy or batch. The calibration test was conducted manually and according to the usual tensile testing method for the Acoustoelastic Constant calibration. As this is a simple, straightforward test, it could easily be performed at the beginning of any construction project and the Acoustoelastic value saved for easier inspections as needed. Further, this test was performed manually and could be replaced by an alternative method such as through the use of a tensioner, which would significantly aid repeatability and give a much more robust value of the Acoustoelastic Constant for further use, and doubles as a loading/unloading fatigue test. It is important to note that this value is unique to every material, and should be performed for every batch of bolts of the same material that is used in the wind turbine assembly, if stress inspections are to be performed on them. One result, at roughly 8.3 MPa, is a significant outlier to the line of best fit. This is likely due to experimental factors, such as a poor couplant coverage.

### 4.3 High stress UT

The results in this section are concerned with a bolt that was put under operational levels of preload. The ultrasonic results are compared in the graph below with the average stress given by the finite element results and the calculated stress using the Acoustoelastic Constant from the section above and the time of flight data. The graph below demonstrates the average stress from the Finite Element Analysis on the *x*-axis and the stress calculated from the ultrasonic time of flight on the *y*-axis. Up to a stress of 75 MPa, the fit is linear as expected. This is shown by the red dashed line in the figure below. However, once past this point, a sudden exponential increase in the calculated stress is seen, represented by the gold line in the below figure.

It is believed that this sudden increase is due to the bolt undergoing plastic deformation. To investigate the reason behind the sudden exponential increase further, the time of flight distribution generated by each of 20 elements of the PAUT probe was plotted. This is shown in Fig. 10 below.

**Fig. 9:**
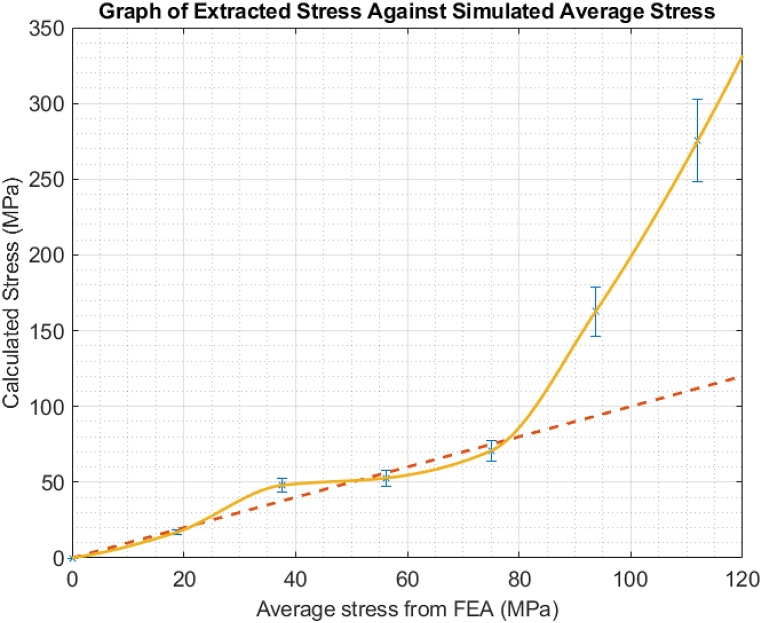
Graph of extracted stress versus measured stress. Note the expected linear fit (dashed) versus the actual result (unbroken).

**Fig. 10:**
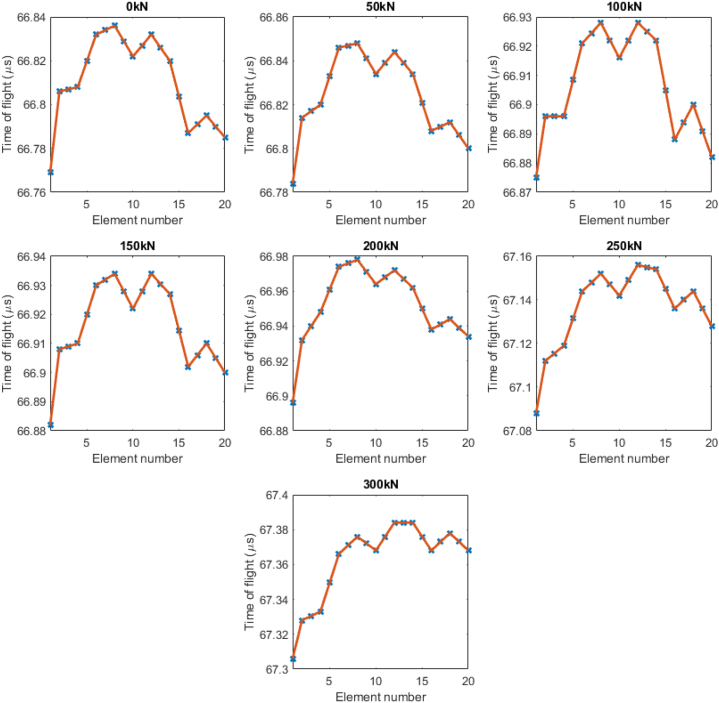
Time of flight distributions for each tightening level. Note that from 200 kN, the symmetry clearly deteriorates.

A key aspect of these results is the demonstration of symmetry deterioration at high loads. Up to a preload of 150 kN, a general symmetry of the Time of Flight distribution is seen about Element 10, which is placed roughly in the centre of the bolt. This begins to degrade at 200 kN and continues to worsen up to 300 kN. This is an unexpected but highly valuable outcome from the multiple element nature of the phased array system, as generating multiple Times of flight and acoustic paths is impossible using a Single Element transducer. It offers a straightforward method of determining if stresses are concentrated on one particular side of a bolt and are likely due to the combined causes of increased stress and extension of the bolt shaft. This is also the likely cause of the sudden exponential increase in stress at the last two points in Fig. 8. This phenomenon arises due to the overall asymmetric nature of bolt tightening, especially when using a hydraulic tensioner because of the slightly different positions of the seating points on the sample. Consequently, achieving a generic assumption of a fully symmetric tensioning process becomes unattainable, particularly on-site. In such instances, where full symmetry is not achieved, the initial tensioning calculations may not be accurately followed. As a result, localized stress concentration and plastic deformation are anticipated in areas experiencing higher stress than the design stress. This difficulty in performing symmetric tensioning, as demonstrated by the unique capabilities of PAUT stress measurement in this experiment, underscores the importance of accounting for and addressing potential asymmetries in the installation of hydraulic tensioners to mitigate the risk of localized stress concentrations and plastic deformation. This issue is ideal for the purposes of this study however, as these bolts would need to be replaced in an operational scenario. Overall, due to the similarity to the current ultrasonic methods the retraining investments of both time and money, though not insignificant, would not be as high as a total inspection methodology switch could be. It is the authors’ belief that the costs would be offset by the savings generated by the more robust stress results and time of flight distribution prediction of deformation, allowing for predictive maintenance and greater levels of certainty regarding whether any given bolt needs to be replaced.

### 4.4 Blind test

The test on the M42 Bolt examined only the unstressed and the operational stress cases, as it was intended to imitate an industrial standard installation. An initial time of flight measurement was obtained and recorded, followed by one when the bolt was torqued to its operative levels. The bolt was tensioned to 440 kN, or around 320 MPa. Due to the length of this particular bolt, variations between unstressed and stressed Time of Flight values were exacerbated, as can be seen in the table below (see Table 1).

**Table 1:** Table comparing calculated and measured results for blind test.

Time of Flight (μs)	Expected Stress from calculation (MPa)	Stress from Ultrasonic Measurement (MPa)
153.21	0	0
155.29	317.5881	352.2922

This is a difference of roughly 2μs in the time of flight, and a measured stress of 352 MPa. This difference is within a 10 % uncertainty and in fact overestimates the stress, which is preferable in industrial contexts to underestimation. This uncertainty could be further improved if an Acoustoelastic calibration could have been performed. The constant from Section 4.1 was used as the bolts were both of marine-grade steel; however, a slightly different value is expected due to the difference in radii and manufacturing conditions. Unfortunately, as the bolt was in use, a full stress gradient as seen in Fig. 9 could not be obtained. In terms of a blind, in situ test, the PAUT system performed well. This cursory test demonstrates the potential of the PAUT system, however further development is required and will be investigated in future work.

## 5 Conclusions

In this paper, a novel methodology for the ultrasonic preload inspection of bolted connections in wind turbines was tested at expected operational loads. The methodology allows for simultaneous defect detection and accurate stress measurement, as well as identification of regions of deformation. A comprehensive study was performed, beginning with a calibration to determine the Acoustoelastic Constant within the bolt material, followed by a test on a clean bolt tightened to operational levels to fully test the methodology as close to an industry standard as possible without travelling to a wind turbine, and finally a blind and in situ test on an M42 bolt. At each stage except the blind test, FEA was performed to obtain an average stress to compare against. The methodology generated expected results, and can additionally identify areas of deformation. This can be used to great effect during any tightening process, and can identify areas where fatigue is causing any deformation over subsequent tests of the same bolts.

Through PAUT path averaging, a better picture of a bolt's true stress can be obtained. While the main advantage here is allowing for replacement of bolts that wouldn't have raised issues before, it also reduces unnecessary replacements. This method allows for more information to be extracted than simply the stress. It can be seen when a bolt has entered the region of plastic deformation (Fig. 9), as the ToF difference from the length extension of the bolt outpaces that due to the Acoustoelastic principle, resulting in the sharp exponential increase. This difference can be seen additionally in the per element ToF distribution, as the symmetrical nature of the results ceases around the deformation window (Fig. 10). The full matrix capture (FMC) technique was unused here, in part to cut down on the processing time. We believe that with access to that volume of data (400 A-scans instead of 20), an even more accurate picture of the stress could be obtained.

In terms of methodology improvements, a further study should be made into the effects of varying temperature and how to account for this within the inspection process. Further, an automatic defect sizing and acoustic path selection algorithm should be considered, to add a further level of efficiency to future inspections.

## Declarations

Data associated with this study has not been deposited into a public repository, and will be made available on request. Review and/or approval by an ethics committee was not needed for this study because no human or animal experiments, or any experiments requiring ethical oversight, were performed.

## CRediT authorship contribution statement

**Brandon Mills:** Writing – review & editing, Writing – original draft, Software, Methodology, Investigation, Formal analysis, Data curation. **Yashar Javadi:** Writing – review & editing, Supervision, Methodology, Investigation, Funding acquisition, Conceptualization. **Farhad Abad:** Writing – review & editing, Software, Investigation, Formal analysis. **Saeid Lotfian:** Supervision, Resources, Project administration, Conceptualization. **Charles MacLeod:** Writing – review & editing, Resources. **Ali Mehmanparast:** Conceptualization. **Gareth Pierce:** Supervision, Resources. **Anthony Gachagan:** Supervision, Resources.

## Declaration of competing interest

The authors declare that they have no known competing financial interests or personal relationships that could have appeared to influence the work reported in this paper.
